# Cannabidiol (CBD): Potential Use in Otorhinolaryngology

**DOI:** 10.1055/s-0043-1777857

**Published:** 2024-02-05

**Authors:** Geraldo Pereira Jotz, Rafael Scorsatto Ortiz, Renata Pereira Limberger, Flavio Anastácio de Oliveira Camargo

**Affiliations:** 1 Innovation and Institutional Affairs, Federal University of Rio Grande do Sul (UFRGS), Porto Alegre, Rio Grande do Sul, Brazil; 2 Pharmaceutical Sciences from UFRGS, Criminology Expert for the Brazilian Federal Government, Porto Alegre, Rio Grande do Sul, Brazil; 3 Federal University of Rio Grande do Sul (UFRGS), Porto Alegre, Rio Grande do Sul, Brazil

Cannabidiol (CBD) is an oily extract from the *Cannabis sativa* plant that is not prohibited, not psychoactive and has been allowed for medicinal use in Brazil since 2015. This cannabinoid has a calming and relaxing effect and is therefore used as an herbal medicine for a wide range of symptoms, particularly in cases of refractory epilepsy. There is also evidence of anticonvulsant, neuroprotective, anti-inflammatory, antitumor, antipsychotic, and anxiolytic effects, as well as effects on functions such as metabolic regulation, movement and coordination, appetite, sleep, mood, pain, reward systems, reasoning and memory, bone growth, and immune function. This cannabinoid has been very prominent in Brazil, in terms both of its use, production, and legislation and of its potential for research and therapy. Despite this evidence, there are few references about the effect of CBD on otorhinolaryngological diseases. This editorial aims to promote understanding and the advancement of studies in this field, with case reports of the use of this cannabinoid in otorhinolaryngology. 1

The main strategy of studies on the potential of CBD relates to cases in which either the existing treatment does not give the desired result or the patient is refractory to treatment. With regard to the former case, **obstructive sleep apnea syndrome** (OSAS) can be highlighted. This is a condition for which traditional pharmacological treatment is limited (Smith et al. 2 ). In the specific case of OSAS, it is possible to consider clinically relevant alternatives for treatment, such as the use of CBD, because, in addition to the complexity of neurochemical control and neuromodulation of the central respiratory drive and upper airway motor output (Carley & Radulovacki 3 ), there are poor tolerance and adherence to long-term treatment with continuous positive airway pressure (CPAP) (Weaver & Grunstein 4 ). The results indicate a strong neuromodulatory role of endocannabinoids in cardiorespiratory autonomic functions, which can be distinguished based on location (Padley et al. 5 ), interactions with other neurotransmitters relevant to sleep–wake behaviors (Di Marzo et al. 6 ), and possibly specific receptor subtypes (Lin and Lee 7 ; Prasad et al. 8 ).

There have also been reports on the use of CBD to relieve **chronic pain** , reduce anxiety, reduce the risk of epilepsy, and even reduce dependence on cigarettes. The analgesic effects help to reduce chronic pain, including neuropathic pain, pain due to spinal cord injuries, arthritis, muscle pain, and even painful syndromes caused by endometriosis, among other types of chronic pain. Cannabinoids can act on various types of pain, in all its complexity, since cannabinoid receptors are widespread across numerous pain-modulating pathways, such as central and peripheral sensory neurons, brain regions that modulate sensory discrimination, pain-regulating circuits in the brainstem, and affective states that regulate emotional responses to noxious stimuli, for example. Endocannabinoids in the body can respond to pain by unblocking one or more of the earliest therapeutically available pathways for obtaining analgesic effects. Clinical data suggest adequate analgesia in chronic (non-malignant) pain. The trial results also suggest that the analgesic efficacy in chronic pain of various causes is probably modulated by all three cannabis chemotypes. The first research results indicate a possible synergy between CBD, tetrahydrocannabinol (THC), and opiates, potentially allowing for combined analgesic effects, while reducing opiate dosages and the risk of adverse effects. 9

The current body of literature suggests that more research is needed to establish the clinical usefulness of CBD in the treatment of chronic pain. It is therefore crucial that physicians consider the fast-track protocol for patients who have an urgent need for pain control and palliation, with a history of significant previous cannabis use, and who may use THC if CBD alone is insufficient to achieve the desired treatment outcome in the standard conservative protocol. Despite the limited clinical evidence supporting the efficacy of CBD in the treatment of chronic pain, safety should always be considered when making treatment decisions. A recent meta-analysis of randomized clinical trials found that the use of nabilone, a synthetic form of THC, was not statistically associated with more adverse events than CBD. However, only 4.5% of all CBD trials reported serious or severe adverse events, compared with 23% of all nabilone trials, further supporting the perception that THC is generally not as well tolerated as CBD. Therefore, starting treatment with CBD-predominant products, especially for patients considered “fragile” or less tolerant, and only introducing THC–CBD balanced products in more severe cases or for “intensive users” can be considered a conservative treatment approach, prioritizing safety considerations over efficacy. 10

Another well-known example of the use of cannabis is in the treatment of **nausea and vomiting** caused by chemotherapy and radiotherapy, improving the patient's quality of life. In the literature, cannabinoids have been shown to be more effective than other antiemetics such as metoclopramide, chlorpromazine, thiethylperazine, haloperidol, domperidone, or alizapride. More recent studies have also examined the ability of cannabinoids, especially CBD, to significantly reduce oxidative stress, inflammation, and cell death induced in the kidneys by chemotherapy, improving kidney function. In general, cannabinoids have been shown to protect organs in many similar ways when chemotoxic exposure is involved. It has also been observed that CBD attenuated THC-induced nausea, as well as the THC-induced increase in corticosteroid use. 11 12

Studies suggest that specific cannabinoids (particularly THC and, to a lesser extent, CBD), the endocannabinoid anandamide, synthetic cannabinoids (e.g., JWH-133), as well as certain terpenes (e.g., eucalyptol), can be used in the therapy and modulation of **cough** , although providing different results through different mechanisms involving CB _1_ , CB _2_ , TRPV1, or FAAH receptors. Cannabis has been shown to have bronchodilator, anti-inflammatory, and antitussive activity on the airways, but there is limited information on the active cannabinoids, their receptors, and the mechanisms for these effects. 13 14

Cannabis products containing exclusively *Cannabis sativa* plant derivatives or phytopharmaceuticals as active ingredients must predominantly contain CBD and no more than 0.2% THC. However, the THC content may exceed 0.2% if the product is intended solely for the palliative care of patients with no other therapeutic alternatives and in clinically irreversible or terminal situations. 15 16

CBD has applications in more than a hundred diseases, signs, and symptoms, as can be found on the CannaKeys platform. 17 This platform ( https://cannakeys.com ) was created to bring together all the results of research involving cannabinoids, with the aim of making available to the population all the published science, critical data, and guidance, so that professionals and patients can obtain the best results in the most efficient way. This database of all the scientific literature on the medicinal use of cannabis-based products consolidates the already widespread understanding of the use of CBD, for example, as a medical treatment with great therapeutic potential in neurodegenerative and psychiatric diseases.
